# The way to relieve college students’ academic stress: the influence mechanism of sports interest and sports atmosphere

**DOI:** 10.1186/s40359-024-01819-1

**Published:** 2024-06-04

**Authors:** Mengfan Liu, Bo Shi, Xu Gao

**Affiliations:** 1 School of Psychology, Northeast Normal University, Changchun, China; 2 School of Physical Education, Northeast Normal University, Changchun, China

**Keywords:** Sports interest, Sports atmosphere, Psychological resilience, Academic stress, Structural equation modeling

## Abstract

**Background and research objectives:**

Given the enduring popularity of higher education, there has been considerable attention on the correlation between college students’ engagement in sports and their academic stress levels. This study seeks to delve deeply into how university physical education fosters academic performance by influencing students’ sports interests, particularly in enhancing their psychological resilience to mitigate academic pressure. Through this investigation, the aim is to offer both theoretical underpinnings and empirical evidence to support the holistic enhancement of higher education.

**Research methods:**

Initially, this study undertakes an analysis of the fundamental relationship between college students’ physical activities and their experience of academic stress. Subsequently, utilizing a structural equation model, specific research models and hypotheses are formulated. These are then examined in detail through the questionnaire method to elucidate the mechanism by which college sports interests alleviate academic stress.

**Research findings:**

The study reveals a significant positive correlation between psychological resilience and academic stress, indicating that a robust psychological resilience can effectively diminish academic pressure. Furthermore, both the sports atmosphere and sports interest are found to exert a notable positive impact on academic stress, mediated by the variable of psychological toughness. This underscores the pivotal role of physical education in fostering positive psychological traits and enhancing academic achievement.

**Conclusion:**

This study underscores the central importance of cultivating and nurturing college students’ sports interests, as well as fostering a conducive sports atmosphere, in fortifying psychological resilience and mitigating academic pressure. By offering novel perspectives and strategies for alleviating the academic stress faced by college students, this study contributes valuable theoretical insights and practical experiences to the broader development of higher education.

## Introduction

In the context of the rapid evolution of higher education, there is a growing focus on enhancing academic resilience to stress [1]. Numerous studies have demonstrated that physical activity serves as a positive psychological intervention, with significant effects on fostering learning, alleviating academic pressure, and reducing psychological anxiety. Given that college students represent the future workforce, their academic stress has garnered widespread concern [2]. Thus, investigating the link between college students’ sports interests and academic pressure holds considerable practical significance and social value. Research on the correlation between sports interests and academic stress has made considerable strides in recent decades. However, most studies have primarily examined their direct relationship, overlooking potential additional factors and complex interactive mechanisms [3]. In recent years, researchers have increasingly turned their attention to the moderating role of mediating variables in the relationship between sports interests and academic stress, such as self-esteem, self-efficacy, and body image. These variables are viewed as pivotal factors influencing the impact of sports interests on academic stress. This study aims to construct a comprehensive theoretical framework and delve deeply into the mechanisms of these mediating variables in the relationship between college students’ sports interests and academic pressure. The goal is to offer more effective strategies and recommendations for alleviating academic pressure among college students.

With the proliferation of higher education and the intensifying social competition, the academic pressure confronting college students has become increasingly pronounced. This pressure not only jeopardizes their mental well-being but also has the potential to impede their academic performance and future career prospects. In recent years, physical activities have garnered widespread attention as an effective means of alleviating academic stress. Research conducted by Karagiorgakis and Blaker [4] underscores the efficacy of sports participation in relieving academic stress among college students. Their findings reveal a significant reduction in anxiety and depression levels among students engaging in sports activities, particularly for those with sustained participation. This discovery presents a positive coping strategy for students to mitigate the psychological stress induced by academic studies through sports engagement. Further evidence from Dexter et al. [5] corroborates the beneficial impact of physical activity in managing academic stress. Their study indicates that students who regularly partake in physical activities demonstrate heightened mental resilience when confronted with academic pressure. This enhanced mental fortitude enables them to confront challenges more adeptly and mitigate negative emotions associated with stress. Approaching the issue from a different angle, O’Connor [6] explores the psychological benefits of sports engagement in alleviating academic stress. He contends that participation in sports fosters feelings of enjoyment and accomplishment, thereby mitigating negative emotions stemming from academic pressure. This sense of fulfillment contributes to heightened life satisfaction and self-confidence among college students, empowering them to confront academic challenges proactively. Storm and Eske [7] emphasize the role of physical activities in nurturing college students’ capacity to cope with challenges and stress. They argue that sports participation equips students with essential coping skills, vital not only for navigating current academic pressures but also for addressing future life and work challenges. From a social standpoint, Green et al. [8] highlight the role of sports participation in fostering social connections among college students. Their research indicates that engagement in sports facilitates the formation of friendships and social networks, providing crucial social and emotional support. This support network aids in mitigating feelings of isolation and helplessness, thereby bolstering students’ psychological resilience in the face of academic stress. Despite the considerable role of physical activity in mitigating academic stress among college students, current research on the relationship between sports interest and academic stress remains limited. Berdida and Grande [9] underscore the growing societal concern regarding the pressure faced by college students, emphasizing the importance of delving deeper into the impact and mechanisms of sports interest on academic stress. Moreover, Basri et al. [10] emphasize the significance of mental health issues among college students, particularly those facing financial hardships, who may experience elevated stress levels and associated psychological challenges. In conclusion, physical activities serve as a potent positive psychological intervention in alleviating the academic stress experienced by college students. Future research endeavors should further explore the impact and mechanisms of sports interest on academic stress, providing both theoretical foundations and practical guidance for mental health education and psychological intervention among college students. This concerted effort will facilitate a better understanding and management of academic stress issues, promoting holistic development and well-being among college students.

Hence, this study endeavors to furnish both theoretical groundwork and practical directives for alleviating academic stress among college students by conducting a systematic review and meta-analysis of extant research. Additionally, it aims to furnish references and insights for prospective investigations in this domain. The primary focus of this inquiry encompasses two key aspects: (1) scrutinizing the relationship between college students’ engagement in sports activities and their academic stress levels, and (2) exploring the moderating variables that influence this relationship. Through a comprehensive examination of pertinent literature and the adoption of both quantitative and qualitative methodologies, this study aims to elucidate these facets. Consequently, it not only furnishes valuable support for ameliorating academic stress among university students but also constitutes a substantial contribution to the advancement of higher education.

### Relationship between college students’ sports interest and academic stress

#### Factors influencing academic stress

Academic stress represents a primary challenge encountered by college students, emanating from diverse sources including coursework demands, examinations, paper submissions, internships, and employment responsibilities [11]. Prolonged exposure to excessive academic stress can precipitate a range of issues such as anxiety, depression, insomnia, and physical discomfort, exerting adverse effects on the mental and physical well-being of college students. Consequently, it is imperative to comprehend the factors contributing to academic stress and to institute tailored coping mechanisms accordingly [12].

Currently, research on academic stress primarily examines individual and environmental factors. Individual factors encompass abilities, interests, and self-expectations, while environmental factors encompass familial, school, and societal pressures. Additionally, factors such as sports interest, sports atmosphere, and psychological resilience play crucial roles in influencing academic stress [13]. Sports interest refers to an individual’s favorable disposition and inclination towards participating in sports activities [14]. College students who harbor sports interests are more predisposed to actively partake in sports, thereby mitigating stress and alleviating anxiety. Research indicates a noteworthy positive correlation between sports interest and mental health status—heightened sports interest correlates with improved mental well-being. Sports atmosphere pertains to the milieu and ambiance surrounding sports activities, encompassing both school and community environments. A conducive sports atmosphere can invigorate college students’ enthusiasm for engaging in sports, thereby enhancing their sports interest and involvement. Studies suggest that an enhanced school sports atmosphere correlates with better mental health among students [15]. Psychological resilience refers to an individual’s capacity to adapt and cope effectively with setbacks, stressors, and adversity [16]. College students endowed with higher levels of psychological resilience demonstrate adeptness in managing academic stress and other pressures, thereby sustaining a positive outlook and fostering good mental health. Research indicates that psychological resilience exerts a significant ameliorative effect on academic stress.

**Fig. 1:**
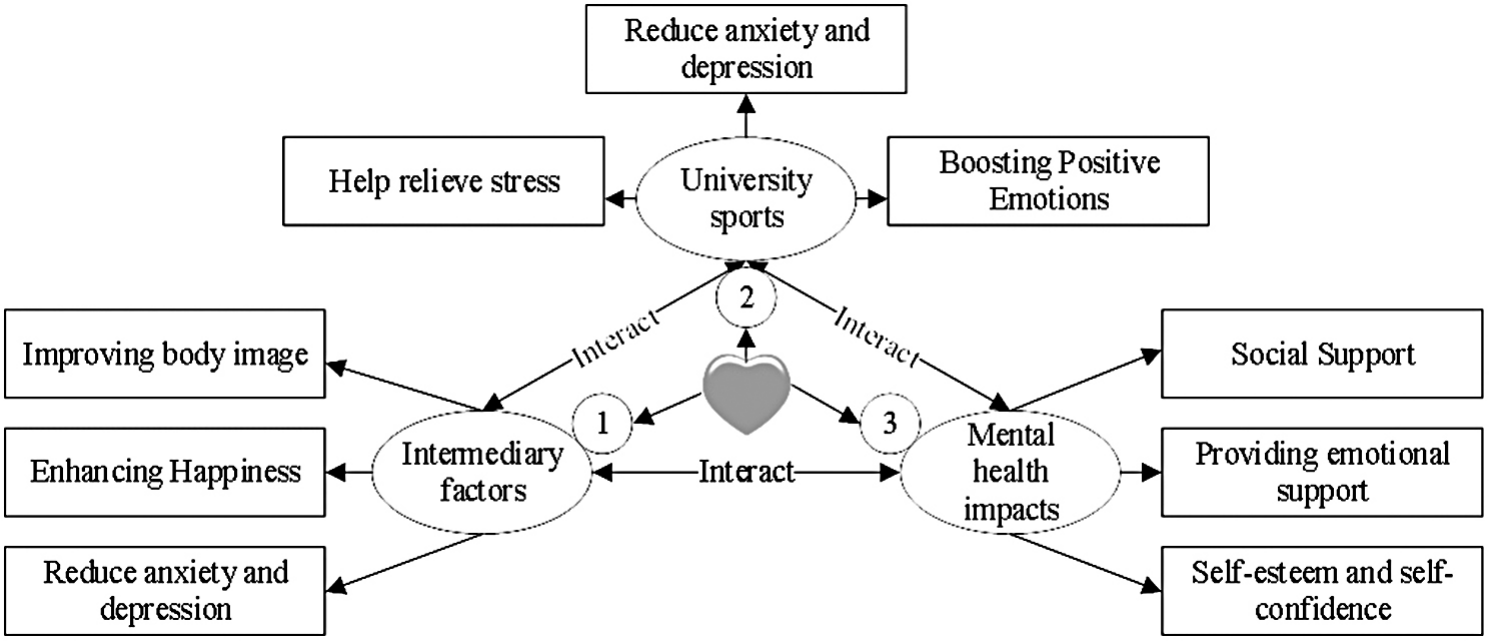
Schematic diagram of the relationship between college students’ mental health and sports activities

In summary, academic stress emerges as a complex issue influenced by various factors. Among these, sports interest, sports atmosphere, and psychological resilience play pivotal roles in shaping the academic stress experienced by college students. The study advocates for the implementation of strategies aimed at enhancing college students’ sports interests, fostering a positive sports atmosphere, and nurturing psychological resilience. These measures are deemed effective in alleviating academic stress and enhancing the mental and physical well-being of college students. Consequently, within the realm of higher education, emphasis should be placed on cultivating students’ sports interests and psychological resilience, while also fostering a conducive sports environment and atmosphere. Figure 1 illustrates the interrelationships between the variables examined in this study.

Figure 1 depicts the positive impact pathway of sports activities on the mental health of college students. Firstly, sports activities contribute to stress relief by triggering the release of substances such as endorphins and dopamine, thereby alleviating anxiety, depression, and fostering positive emotions [17]. Secondly, participation in sports bolsters college students’ self-esteem and confidence. Through showcasing their abilities and achieving success, students garner recognition and affirmation of their self-worth. Additionally, forging close connections with fellow athletes facilitates the formation of a supportive social network, offering emotional reinforcement and encouragement. Consequently, this diminishes feelings of social isolation and loneliness, augmenting happiness and mental well-being [18]. Moreover, engagement in sports endeavors can enhance body image and satisfaction, fostering increased self-acceptance and contentment with one’s physical appearance, thereby mitigating anxiety and stress associated with appearance. Importantly, the relationship between college students’ sports activities and mental health is reciprocal: active involvement in sports enhances mental well-being, while robust mental health status further motivates students to participate actively in sports. Hence, actively promoting campus sports activities emerges as a potent means to nurture the mental health of college students [19].

## Application of structural equation models

Structural equation models (SEMs) stand as a robust statistical technique extensively employed in social science research, particularly for elucidating intricate causal relationships and latent variables. In this study, SEMs are utilized to delve deeply into the interplay between college students’ sports interests, sports atmosphere, and academic stress, while also examining the potential role of psychological resilience. Through SEMs, interactions among multiple variables can be simultaneously estimated, encompassing the direct effects of independent variables (e.g., sports interest and sports atmosphere) on dependent variables (e.g., academic stress), as well as their indirect effects mediated through latent variables (e.g., psychological resilience) [20]. This comprehensive analytical approach fosters a more precise comprehension of the complex relationships among these variables and their collective impact on college students’ academic stress and mental health. It is noteworthy that although sports interest and sports atmosphere serve as independent variables, they do not directly function as moderators. Moderating factors typically denote variables that can influence the relationship between independent and dependent variables, such as gender, age, or cultural background. For instance, gender can be regarded as a moderating factor in research aimed at exploring disparities between different gender groups regarding the influence of sports interest and sports atmosphere on academic stress. By introducing gender as a moderator, researchers can attain a more holistic understanding of whether the relationship between college students’ sports interest, sports atmosphere, and academic stress varies across genders. This, in turn, facilitates the development of tailored educational and intervention measures to better cater to the diverse needs of different gender groups and promote their holistic development [21].

**Fig. 2:**
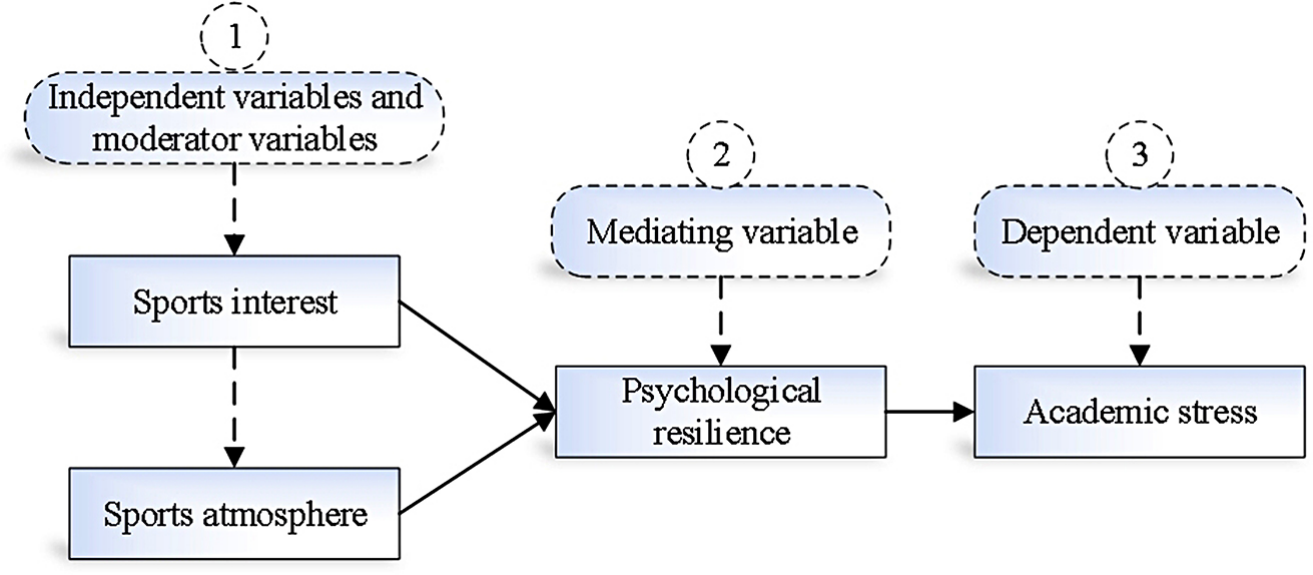
Relationship of study variables

Figure 2 illustrates the design of variable relationships in this study, based on the preceding discussion.

The research hypotheses for this study are as follows:

### H1:

Sports interest has a direct positive impact on psychological resilience.

### H2:

Sports interest has an indirect positive impact on academic stress through psychological resilience.

### H3:

Sports atmosphere has a direct positive impact on psychological resilience.

### H4:

Sports atmosphere has an indirect positive impact on academic stress through psychological resilience.

Through the utilization of SEMs, this study can effectively test these hypotheses and estimate the effects of each pathway [22]. This facilitates a comprehensive understanding of the intricate relationships among sports interest, sports environment, psychological resilience, and academic stress. Moreover, it furnishes a theoretical foundation for intervening and preventing psychological health issues among college students [23].

While SEMs offer a potent tool for investigating complex relationships, they are not without challenges and limitations [24]. For example, SEMs can solely manage known and pre-defined variables and are unable to accommodate unknown or undefined variables [25]. Additionally, the hypothesis-testing nature of SEMs necessitates cautious interpretation of the results in this study. To mitigate these limitations and ensure the reliability of results, this study employs an appropriate sample size and relied on reliable measurement tools for assessing variables [26].

## Research methods

This study predominantly employs a questionnaire survey method to gather data to thoroughly investigate the relationship between college students’ sports interests, sports atmosphere, psychological resilience, and academic pressure. The questionnaire design encompasses four primary sections: basic information of college students, sports interests, sports atmosphere, and psychological resilience, and academic pressure.

(1) Questionnaire design

To ensure the scientific rigor and validity of the questionnaire, this study opted for four widely validated scales:

Athletic Climate Scale: Developed by Jackson and Marsh in 1996, this scale evaluates college students’ experiences in athletic environments. It comprises 8 single-dimensional indicators and is scored on a 10-point scale [27].

Intrinsic Motivation Scale: This scale, devised by Ryan and Deci in 2000, gauges college students’ interest in physical activities. It consists of 8 indicators and is scored on a 7-point scale [28].

Connor-Davidson Resilience Scale: Developed by Connor and Davidson in 2003, this scale assesses an individual’s psychological resilience. It encompasses 25 indicators and is scored on a 5-point scale [29].

Academic Stress Scale: Based on Lefcott’s 1981 study, this scale is utilized to evaluate college students’ experience of academic stress. It includes 15 indicators, also scored on a 5-point scale [30].

(2) Sampling method

To ensure the breadth and representativeness of the sample, this study meticulously designs and implements the sampling strategy. Initially, the target groups are meticulously segmented based on multiple dimensions such as geographical location, school type, and subject area, thereby forming multiple levels or subgroups with distinct characteristics. This segmentation facilitates a more comprehensive coverage of college students from diverse backgrounds, thereby ensuring the diversity of the sample. Subsequently, within each subpopulation, a random sampling method is employed to ensure that individuals in each subgroup have an equal opportunity of being selected. This randomness effectively mitigates sampling errors and enhances the representativeness of the sample. Through random sampling, it is ensured that individuals in the sample are sufficiently dispersed across geographical locations, school types, and subject areas, thus authentically reflecting the overall scenario of the target group. Furthermore, clear inclusion criteria are established to ensure homogeneity of the sample and the reliability of the study. Specifically, survey participants are required to be full-time college students, ensuring uniformity in educational backgrounds and experiences. This inclusion criterion serves to control for confounding factors within the sample and bolster the internal validity of the study. By employing stratified random sampling and setting explicit inclusion criteria, this study successfully constructs a broad and representative sample, laying a robust foundation for subsequent data analysis and the dissemination of research findings.

(3) Data collection and processing

**Fig. 3:**
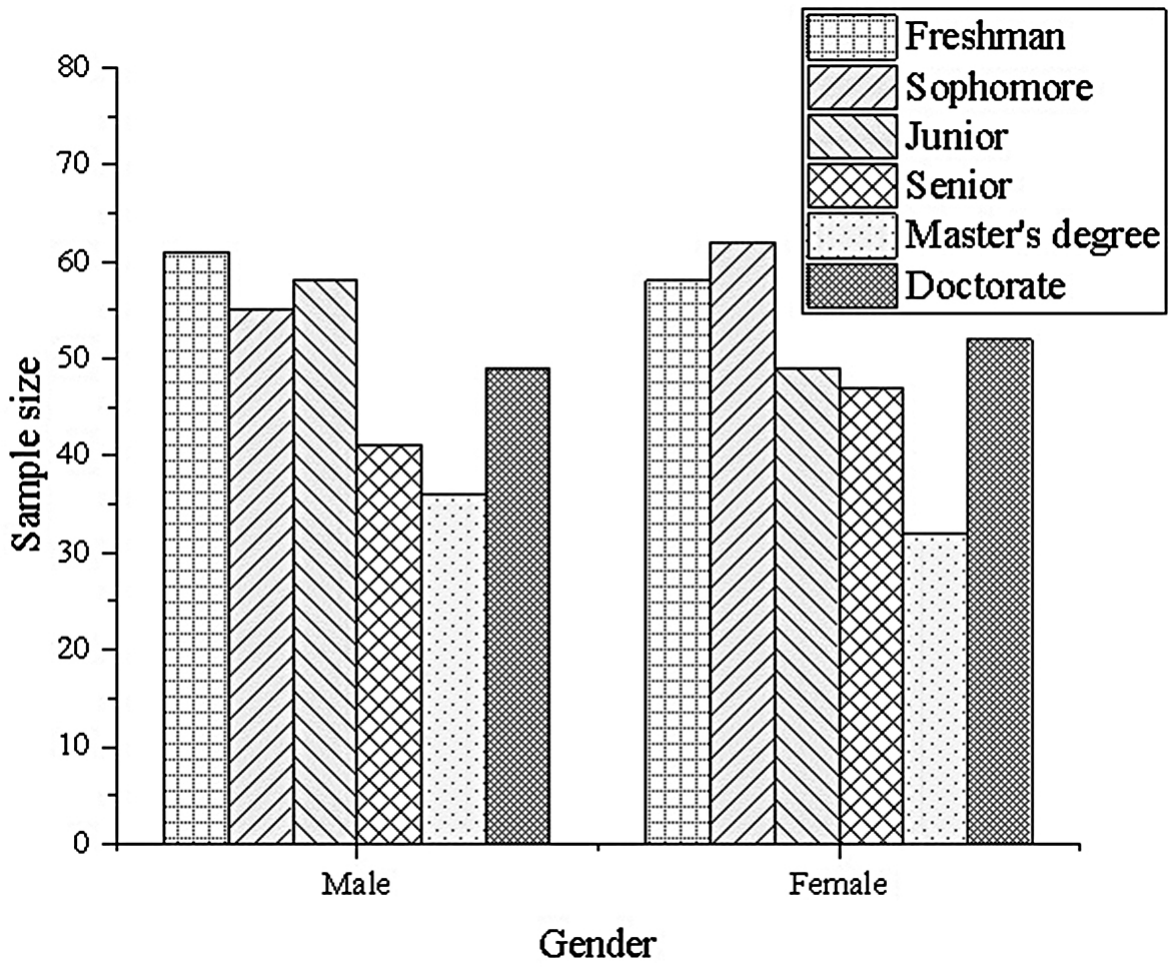
Sample statistical results

Through meticulously planned online survey initiatives, 725 questionnaires are successfully distributed across major online survey platforms. These questionnaires are widely circulated among university students from diverse regions, age groups, and academic backgrounds, ensuring the diversity and representativeness of respondents. Following the collection of questionnaires, rigorous screening and sorting procedures are implemented to eliminate incomplete or erroneous responses, as well as those that do not meet the predefined inclusion criteria, thereby ensuring the validity and reliability of the data. Subsequently, after this thorough screening and sorting process, a total of 706 valid questionnaires are retained, yielding an effective rate of 97.38%. This high retention rate underscores the rationality of the questionnaire design and the active cooperation of the respondents. The respondents to these valid questionnaires hail from various regions across the country, spanning diverse age groups and academic disciplines. Their responses furnish a rich database, facilitating a comprehensive and in-depth understanding of college students’ sports interests, sports atmosphere, psychological resilience, and academic pressure, among other aspects. Upon completion of data collection, the collected data undergo detailed analysis utilizing SPSS statistical software. Initially, descriptive statistics are conducted to delineate the distribution of each variable, providing a preliminary insight into the overall sample characteristics. Subsequently, correlation analysis is performed to explore the interrelationships between variables, laying the groundwork for subsequent regression analysis. Finally, regression analysis and other analytical techniques are employed to delve deeply into the relationships and influencing mechanisms among variables such as sports interest, sports atmosphere, psychological resilience, and academic pressure, furnishing robust evidence for understanding the intricate interplay among these variables. The specific outcomes of the statistical analysis are elucidated in Fig. 3.

As depicted in Fig. 3, the research sample of this study comprises 706 college students, 363 girls and 343 boys. Furthermore, the sample encompasses students from all academic years within the university, indicating that the research sample encompasses the entire student body on campus. This comprehensive coverage of the student population imbues the research findings with significant practical relevance and applicability.

The defined results of variables in this study are as follows:

Sports atmosphere comprises three subclassifications: Cooperation (M1), Input (M2), and Sense of belonging (M3). Sports interests contains 3 subclassifications: Self(I1), Mode (I2), and Participate (I3). Psychological resilience contains 4 subclassifications: Self-regulation (R1), Help from others (R2), and Ability dictates (R3), Learning effect (R4). Academic stress contains 4 subclassifications: Influence of others (P1), Competitive Impact (P2), Ability Impact (P3), and Environmental impact (P4).

In summary, this study process meticulously defines key variables, including sports interest, sports atmosphere, mental toughness, and academic stress. Sports interest is gauged through pertinent questions in the questionnaire, reflecting individuals’ preferences and willingness to engage in sports activities. The sports climate is evaluated using a specialized sports climate scale, elucidating the extent to which an individual’s sports environment or community fosters and supports sports participation. Mental toughness is assessed employing the well-established Resilience Scale, capturing an individual’s capacity to adapt and rebound amidst challenges and adversity. Academic stress is quantified using the Academic Stress Scale, focusing on the intensity of pressure and tension experienced by students during the learning process. To ensure the accuracy and reliability of these variables, the study conducts a thorough literature review, selects validated scales, and performs factor analysis as necessary to delve deeper into the structure and dimensions of the variables. These variables play pivotal roles in the study, and understanding their interactions and collective influence on an individual’s psychological state and academic performance constitutes the core content of the investigation. Research inquiries centers on elucidating the interrelationships among these variables and how they synergistically shape an individual’s holistic development. Drawing from theoretical analysis and literature review, the research team formulates a series of hypotheses to be tested through data collection and analysis. By meticulously measuring and analyzing these variables, this study aims to offer a nuanced understanding of the nexus between sports interest, sports atmosphere, mental toughness, and academic pressure, furnishing valuable insights for both research and practical applications in related fields.

### Analysis of the impact of college students’ sports on mental health

#### Questionnaire reliability analysis

Reliability pertains to the consistency and stability of measurement results obtained from a questionnaire, constituting a crucial criterion for evaluating questionnaire quality. Various methods exist for assessing reliability, with test-retest reliability, split-half reliability, and Cronbach’s Alpha being commonly utilized tools. Among these, Cronbach’s Alpha coefficient method has emerged as one of the most prevalent reliability analysis techniques due to its simplicity and effectiveness. It assesses the internal consistency of a questionnaire by computing the correlation between all items within the questionnaire. Typically, a Cronbach’s Alpha coefficient exceeding 0.7 signifies good reliability of the questionnaire.

In this study, the reliability of the questionnaire is evaluated using Cronbach’s Alpha coefficient method. The analysis reveals a Cronbach’s Alpha coefficient of 0.961, significantly surpassing the generally accepted threshold for good reliability (0.7). This outcome unequivocally indicates that the questionnaire employed here exhibits exceptionally high internal consistency, implying that each item within the questionnaire demonstrates strong consistency when measuring the same construct. Thus, the questionnaire can be deemed to possess good reliability.

Furthermore, to ascertain the suitability of the questionnaire for further factor analysis, a Kaiser-Meyer-Olkin (KMO) test is conducted. The KMO test evaluates the adequacy of raw data for factor analysis by assessing the ratio of partial correlations to simple correlations between variables. A KMO value approaching 1 signifies strong correlation between variables, rendering the original variables more conducive for factor analysis. Conversely, a KMO value nearing 0 suggests weak correlation between variables, rendering the original variables unsuitable for factor analysis.

In this study, the KMO test yields a result of 0.975, indicating a very high value close to 1, signifying robust correlation between variables in the questionnaire. Consequently, it can be inferred that the questionnaire utilized in this study is highly suitable for factor analysis, thereby further validating the reliability and applicability of the questionnaire. In summary, based on the aforementioned analysis, it can be concluded that the questionnaire employed in this study exhibits both good reliability and good validity.

#### Influence of personal factors

When assessing the influence of gender, grade, place of origin, and academic performance on college students’ engagement in physical exercise and mental health, this study employs the following statistical methods:

For examining the relationship between categorical variables such as gender, grade, place of origin, participation in physical exercise, and mental health level, a Chi-square test is utilized. This method is employed to ascertain whether the observed frequency distribution across different categories significantly differs from the expected frequency distribution, thereby determining the presence of a correlation between two categorical variables.

Moreover, one-way ANOVA is employed for assessing the relationship between the continuous variable of academic performance and physical exercise participation and mental health levels. This statistical method is utilized to examine the effect of one or more independent variables (in this study, academic performance) on a continuous dependent variable (such as physical activity participation or mental health levels). Through the application of the aforementioned statistical methods, the results presented in Table 1 are obtained. This table provides a comprehensive overview of the statistical analysis outcomes concerning gender, grade, place of origin, academic performance, physical exercise participation, and mental health level.

**Table 1:** Impact of personal influencing factors

Title	Gender		Grade		Home location		Score	
	*χ2*	*p*	*χ2*	*p*	*χ2*	*p*	*χ2*	*p*
Sports atmosphere	0.421	0.517	6.127	0.106	0.717	0.397	5.134	0.274
Sports interests	12.463	0.052	43.325	0.001**	5.448	0.448	15.461	0.907
Psychological resilience	17.559	0.002**	10.610	0.563	5.248	0.263	14.315	0.575
Academic stress	6.618	0.157	9.990	0.617	4.413	0.353	19.430	0.247

* *p* < 0.05 ** *p* < 0.01.

The findings depicted in Table 1 reveal that only gender demonstrates a significant correlation with psychological resilience, while grade level exhibits a noteworthy relationship with sports interest. Moreover, throughout the testing process, the influence of gender, grade level, hometown, and academic performance on the variables under investigation in this study is deemed insignificant. These results suggest that external factors exert minimal impact on the study, thereby affirming its reliability.

#### Correlation analysis

This study analyzes the relationship between various variables using the Pearson correlation coefficient to examine the linear relationships within the model. The Pearson correlation coefficient is a statistic that measures the strength and direction of the linear relationship between two variables, ranging from − 1 to 1. A correlation coefficient close to 1 indicates a strong positive correlation, close to -1 indicates a strong negative correlation, and close to 0 indicates almost no linear relationship between the variables. By calculating the Pearson correlation coefficient for the relevant variables in the dataset, the intensity and direction of the linear relationship between them are obtained. The results are summarized in Table 2, providing an important basis for further analysis of interactions between variables and the construction of statistical models.

**Table 2:** Test of linear correlation

	Unstandardized coefficient		Standardized coefficient	t	*p*	Collinearity diagnosis	
	B	Standard error	Beta			VIF	Tolerance
Constant	1.403	0.268	-	5.225	0.000**	-	-
Sports interests	-0.042	0.059	-0.087	-0.709	0.480	1.573	0.636
Psychological resilience	0.078	0.044	0.177	1.773	0.079	1.042	0.959
Academic stress	-0.041	0.036	-0.139	-1.141	0.257	1.568	0.638
*R* ^2^	0.087						
Adjustment *R*^2^	0.058						
*F*	*F* (3,96) = 3.042, *p* = 0.033						
D-W value	1.710						

*p < 0.05 **p < 0.01.

The results presented in Table 2 from the linear correlation test shed light on the relationships between sports interest, psychological resilience, academic pressure, and psychological well-being. While sports atmosphere is not directly listed, its impact on psychological well-being is discussed based on the provided description. These tests offer crucial insights into how these variables interrelate. The unstandardized coefficient for sports interest is -0.042, indicating that increased sports interest is associated with a small yet significant positive effect on psychological well-being. Although the standardized coefficient Beta is -0.087 and the t value is -0.709 (low), the p value is 0.480 (greater than 0.05 significance level), suggesting that the direct correlation between sports interest and psychological well-being is not strong. However, this doesn’t undermine the importance of sports interests. Conversely, the unstandardized coefficient for psychological resilience is 0.078, indicating that enhancements in psychological resilience are linked to significant positive effects on psychological well-being. With a standardized coefficient Beta of 0.177, a t value of 1.773, and a p value of 0.079 (close to the significance level of 0.05), the role of mental toughness in bolstering mental health is underscored. Moreover, the unstandardized coefficient for academic stress is -0.041, indicating that heightened academic stress is associated with negative effects on psychological well-being. Although the standardized coefficient Beta is -0.139, the t value is -1.141, and the p value is 0.257 (greater than 0.05 significance level), implying that the direct negative impact of academic stress on psychological well-being is not significant, its negative impact trend is noteworthy. Regarding sports atmosphere, although its coefficient is not listed, the p-value is 0.480, indicating its insignificant impact on psychological well-being. This warrants further exploration in future research to discern the potential influence of sports atmosphere on psychological well-being. In terms of analysis of variance, the model’s R² value is 0.087, indicating that sports interest, mental toughness, and academic stress jointly explain approximately 8.7% of the variation in psychological well-being. Though modest, this proportion holds significance given the multifaceted nature of psychological well-being. The adjusted R² value is 0.058, showing a slight decrease in the model’s explanatory power after accounting for the number of variables. With an F statistic value of 3.042 and a corresponding p-value of 0.033 (less than the 0.05 significance level), the entire model is statistically significant in predicting psychological well-being. The Durbin-Watson (D-W) value of 1.710 indicates no evident autocorrelation between residuals, ensuring the model’s data independence. In conclusion, this study elucidates the intricate relationships between sports interest, psychological resilience, academic stress, and psychological well-being. While the direct effect of sports interest on psychological well-being may not be significant, it likely plays a significant role alongside other variables. The substantial positive impact of psychological resilience on psychological well-being offers novel insights into enhancing mental health. Conversely, the negative trend of academic stress on psychological well-being underscores its potential harm. Future research can delve deeper into the interaction mechanisms between these variables and devise interventions to enhance college students’ psychological well-being.

#### Effects of sports on academic stress in the SEMs

**Fig. 4:**
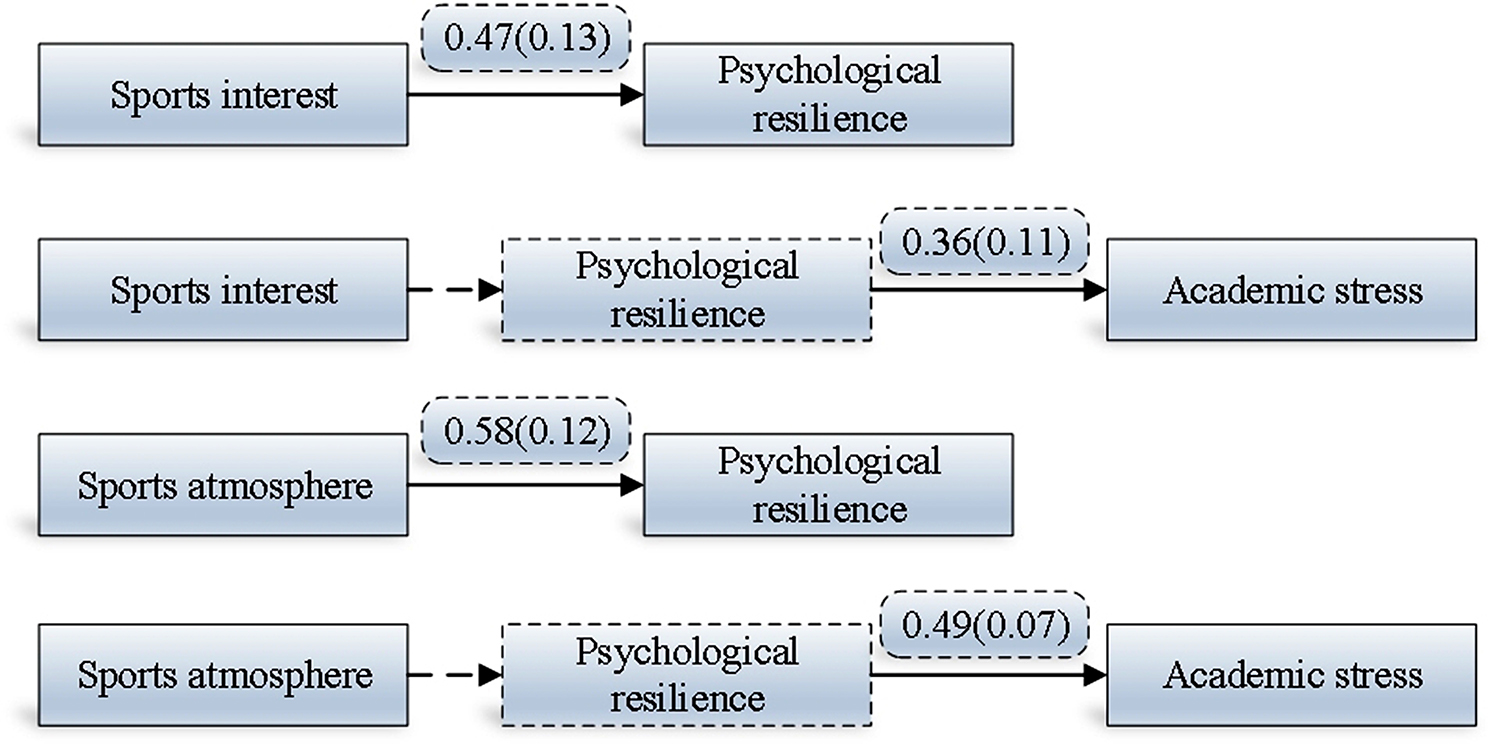
Effects of sports on academic stress in the SEMs

Indeed, correlation analysis and SEMs are robust analytical tools that are wisely chosen for this study to comprehensively evaluate research hypotheses and explore relationships between variables. Correlation analysis serves as an initial step to ascertain statistical correlations between variables, providing a foundation for deeper investigation. By assessing the strength and direction of these correlations, researchers gain insights into potential associations and can identify variables that warrant further examination. SEMs, on the other hand, offers a sophisticated approach to analyze complex relationships among multiple variables simultaneously. It allows researchers to construct models that incorporate both observed variables and latent variables, providing a more nuanced understanding of their interactions and overall impact. SEMs serve as an efficacious statistical approach for probing the association between sports activities and academic stress. This methodology facilitates the examination of both the direct and indirect impacts of various types of sports activities on academic stress, while also considering the influence of individual differences on this nexus. Such an approach not only enhances comprehension of the mechanisms by which sports activities influence academic stress but also furnishes valuable insights for ameliorating academic stress. Table 3; Fig. 4 illustrate the research findings pertaining to the influence of sports activities on academic stress, as explored through SEMs in this study.

**Table 3:** The comprehensive impact of sports on academic stress

Hypothesis	Variable type	Path coefficient	T-value	*P*-value	Hypothesis verification
H1	Sports interest → psychological resilience	0.47(0.13)	5.26	< 0.001	established
H2	Sports interest → Psychological resilience → Academic stress	0.36(0.11)	4.15	< 0.001	established
H3	Sports atmosphere → psychological resilience	0.58(0.12)	5.14	< 0.001	established
H4	Sports atmosphere → Psychological resilience → Academic stress	0.49(0.07)	4.56	< 0.001	established

The path analysis results presented in Table 3; Fig. 4 provide strong support for the research hypotheses, confirming the significant impact of sports interest and sports atmosphere on college students’ psychological resilience and academic stress. Let’s break down these findings based on each hypothesis:

##### H1:

Sports interest has a direct positive impact on psychological resilience.

The path coefficient of 0.47, with a t value of 5.26 and a p value of less than 0.001, indicates a significant positive relationship between sports interest and mental toughness. This result confirms the hypothesis, suggesting that higher levels of sports interest are associated with greater psychological resilience among college students.

##### H2:

Sports interest has an indirect positive impact on academic stress through psychological resilience.

The path coefficient of 0.36, with a t value of 4.15 and a p value of less than 0.001, supports the indirect effect of sports interest on academic stress through psychological resilience. Thus, not only does sports interest directly enhance psychological resilience, but it also indirectly reduces academic stress by bolstering psychological toughness.

##### H3:

Sports atmosphere has a direct positive impact on psychological resilience.

The path coefficient of 0.58, with a t value of 5.14 and a p value of less than 0.001, indicates a significant positive impact of sports atmosphere on psychological resilience. This finding corroborates the hypothesis, suggesting that a positive sports environment fosters greater psychological resilience among college students.

##### H4:

Sports atmosphere has an indirect positive impact on academic stress through psychological resilience.

The path coefficient of 0.49, with a t value of 4.56 and a p value of less than 0.001, provides evidence for the indirect effect of sports atmosphere on academic stress via psychological resilience. Therefore, a conducive sports atmosphere not only enhances psychological resilience but also indirectly mitigates academic stress by bolstering mental toughness.

Absolutely, interpreting path coefficients in SEMs requires careful consideration of various factors that may influence their reliability and validity. Sample size, measurement error, model specification, and data characteristics can all impact the accuracy of path coefficients and the overall fit of the model to the data. Sample size plays a crucial role in the statistical power of SEMs. Larger sample sizes generally provide more reliable estimates of path coefficients and increase the likelihood of detecting significant relationships. Measurement error can introduce bias into path coefficient estimates, leading to potential inaccuracies in model interpretation. Therefore, it is essential to use validated measurement tools and carefully consider the reliability and validity of the measures used in the study. Furthermore, model fit statistics, such as the comparative fit index and root mean square error of approximation, are critical for assessing the overall goodness of fit of the SEM to the data. Poor model fit may indicate discrepancies between the proposed theoretical model and the observed data, highlighting the need for model refinement or reconsideration of data processing methods. Figure 4 provides a visual representation of the path analysis results, offering an intuitive understanding of the relationships between sports atmosphere, sports interest, mental toughness, and academic pressure among college students. This visualization aids in interpreting the complex interplay between these variables and facilitates communication of the study findings. In conclusion, addressing issues related to sports atmosphere, sports interest, mental toughness, and academic pressure among college students requires a multifaceted approach. Strategies such as strengthening physical education programs, providing mental toughness training, optimizing curriculum design, and improving teaching methods can contribute to promoting the physical and mental well-being of college students and equipping them with the skills to navigate the challenges of modern society.

## Discussion

This study delves into the influence of physical exercise on academic stress among college students, with a specific focus on how it shapes students’ psychological resilience and consequently impacts their academic stress levels. In today’s university milieu, characterized by escalating competition, academic stress emerges as a formidable challenge for college students. Hence, investigating the efficacy of physical exercise as a stress-relief mechanism assumes paramount significance in safeguarding the psychological well-being of students. While extant literature extensively examines the correlation between physical exercise and physical health, scant attention has been paid to its ramifications on academic stress and psychological resilience. This study bridges this gap by employing SEMs to meticulously scrutinize the intricate interplay among physical exercise, psychological resilience, and academic stress. This methodological approach not only offers a novel research perspective but also sheds light on the pivotal roles played by sports interest and the sports atmosphere in shaping college students’ psychological resilience and mitigating academic stress. The findings underscore the substantial mediating role of psychological resilience between physical exercise and academic stress. Moreover, they reveal that both sports interest and the sports atmosphere wield a significant positive influence on psychological resilience, and bolstering psychological resilience effectively diminishes academic stress levels among college students. These findings underscore the multifaceted significance of sports in higher education, extending beyond mere physical health promotion to encompass a distinctive role in safeguarding psychological well-being and alleviating academic stress.

This study makes a significant contribution by explicitly elucidating the inherent connections among interest in sports, the sports atmosphere, psychological resilience, and academic stress, thereby underscoring the positive role of sports in maintaining the psychological well-being of college students. This finding not only enriches theoretical understanding in relevant fields but also provides practical strategies and recommendations for schools and educational institutions. Firstly, schools and relevant departments should further recognize the importance of sports in alleviating academic stress and promoting psychological health, intensifying efforts to promote sports activities. By offering diverse sports programs and facilities and encouraging active participation in sports activities among college students, schools can nurture their interest in sports and cultivate healthy sports habits. Secondly, educational institutions and educators should integrate sports into daily educational teaching, making it an essential means of promoting students’ comprehensive development and enhancing psychological resilience. By creating a positive sports atmosphere through various forms such as sports classes, sports clubs, and sports events, educators can help students establish healthy lifestyles. Finally, future research could further explore other factors that may influence the relationship between interest in sports, psychological resilience, and academic stress, such as individual traits, family background, and social environment. This will contribute to a more comprehensive understanding of the role of sports in the growth of college students, providing scientific evidence for the development of more effective intervention measures. In conclusion, this study offers a new perspective on understanding the regulatory mechanism of college students’ psychological health by deeply exploring the relationship between physical exercise, psychological resilience, and academic stress. By fostering interest in sports, enhancing psychological resilience, and creating a positive sports environment, academic stress among college students can be effectively alleviated, promoting their comprehensive development and psychological health. These findings hold significant practical implications for schools, society, and families alike.

## Conclusion

With societal development and the acceleration of life’s pace, academic pressure has garnered widespread attention. Sports interest and a favorable sports atmosphere are increasingly recognized as effective methods for alleviating academic pressure. This study aims to explore the influence of physical activities and psychological resilience among college students on academic stress, offering valuable insights for addressing the academic stress levels of college students. Through a literature review and SEM analysis, this study presents significant conclusions and contributions. Firstly, it establishes a notable relationship between psychological resilience and academic stress, demonstrating that the sports atmosphere and sports interest significantly impact academic stress through psychological resilience. This underscores the importance of fostering sports interests and cultivating a positive sports atmosphere among college students to enhance psychological resilience, consequently alleviating academic pressure. This finding provides vital guidance for college educators, highlighting the importance of organizing and promoting sports activities. Secondly, through SEM analysis, this study identifies psychological toughness as a mediating factor in the relationship between sports interest and academic stress. This underscores the positive impact of physical activity on mental health, specifically in reducing academic stress by enhancing psychological toughness. This contribution enriches the theoretical framework surrounding the interplay of academic stress and physical activity and offers novel perspectives and strategies for mental health education in higher education institutions. Finally, this study validates its research hypotheses through empirical data analysis and offers specific recommendations and future prospects. However, certain limitations exist, including potential subjectivity in self-reporting and memory constraints inherent in the questionnaire method. Thus, future research endeavors may focus on diversifying samples and expanding coverage to enhance the reliability and representativeness of research outcomes. In summary, this study provides valuable insights and strategies for ameliorating the academic stress experienced by college students by delving into the influence of physical activities and psychological resilience on academic stress. This not only aids university educators in better addressing students’ mental health concerns but also serves as a significant reference for future related research.

## Data Availability

The datasets generated and analysed during the current study are not publicly available due to privacy reasons, but are available from the corresponding author on reasonable request.
